# Supplementary material for: Hydrophobic Organic Pollutants in Soils and Dusts at Electronic Waste Recycling Sites: Occurrence and Possible Impacts of Polybrominated Diphenyl Ethers
Source: Int J Environ Res Public Health. 2019 Jan 28;16(3):360. doi: 10.3390/ijerph16030360 (PMC6388296; doi:10.3390/ijerph16030360)
Supplement: Supplementary file 1 [file ijerph-16-00360-s001.pdf]

Supplementary Materials

Table S1a. Summary of the Physicochemical parameters (mean of pH and Total Organic matter content) of the samples at the study locations. No standard deviation is given if n=1.

	**Burning Sites**		**Dismantling Sites**		**Repair Sites**		**Control Sites**	
	**pH**	**TOC%**	**pH**	**TOC%**	**pH**	**TOC%**	**pH**	**TOC%**
Soil	7.8 ± 0.43	20 ± 11	5.8 ± 3	7.4 ± 6.3	8.8	1.5	8.0 ± 0.4	3.2 ± 0.9
Roadside dust	-	-	8.3± 0.06	4.4 ± 2.6	8.1	1.5	8.3 ± 0.5	1.3 ± 0.3
Floor dust	-	-	8.4 ± 0.24	5 ± 1.0	8.4 ± 0.3	4.1 ± 4.2	8.8	5.3
Direct dust	-	-	-	-	9 ± 0.2	17.4 ± 6	-	-

Table S1b. Physicochemical parameters (pH and Total Organic Matter Content) of the samples at the study site.

E-Waste Activity Sites		Lagos				Ibadan				Aba			
		pH	Control pH	%TOC	Control %TOC	pH	Control pH	%TOC	Control %TOC	pH	Control pH	%TOC	Control %TOC
Burning sites													
Soil(0–10cm)	Mean ± SD	7.7	7.9 ± 0.4	36.3	3.5 ± 1.2	8.2 ± 0.2	8.2 ± 0.24	16.8 ± 2.4	3.6 ± 1.6	7.5 ± 0.44	7.5 ± 0.62	16 ± 11.5	2.3 ± 0.7
	Minimum	7.7	7.41	36.3	2.4	8.03	8.1	15.1	2.5	7.21	7.10	7.98	1.8
	Maximum	7.7	8.22	36.3	5.15	8.4	8.4	18.45	4.7	7.84	7.97	24.24	2.8
Dismantling site													
Soil(0–10cm)	Mean ± SD	7.94 ± 0.23	7.9 ± 0.35	11 ± 11.4	3.5 ± 1.2	5 ± 2	8.2 ± 0.24	5.8 ± 2.5					
	Minimum	7.74	7.41	1.58	2.4	1.94	8.07	2.74					
	Maximum	8.2	8.22	23.69	5.15	9.0	8.41	10.3					
Floor dust	Mean ± SD	8.4 ± 0.24		4.9 ± 1									
	Minimum	8.14		3.71									
	Maximum	8.7		6.19									
Direct dust	Mean ± SD												
	Minimum												
	Maximum												
Roadside dust	Mean ± SD	8.3 ± 0.6	8.2 ± 0.6	4.4 ± 2.6	1.2 ± 0.3					8.06	8.15 ± 0.6	1.51	1.3 ± 0.35
	Minimum	8.25	7.6	2.61	0.98					8.06	7.1	1.51	0.6
	Maximum	8.34	8.7	6.24	1.6					8.06	9.0	1.51	1.7
Repair site													
Floor dust	Mean ± SD	8.4 ± 0.6		3.4 ± 0.95		8.6 ± 0.4	8.8 ± 8.8	8.3 ± 7.9	5.3 ± 5.3	8.2 ± 0.25		2.4 ± 0.83	
	Minimum	8		1.85		8.22	8.8	3.4	5.3	7.95		1.14	
	Maximum	9		5.01		8.96	8.8	22.33	5.3	8.8		3.86	
Direct dust	Mean ± SD					9 ± 0.2		17.4 ± 5.6					
	Minimum					8.8		10					
	Maximum					9.24		22.4					
Roadside dust	Mean ± SD									8.1	8.4 ± 0.5	1.5	1.4 ± 0.3
	Minimum									8.1	7.8	1.5	0.99
	Maximum									8.1	9.0	1.5	1.7

Table S2. PBDE median concentrations (ng/g dry weight) across various e-waste sites; and exceedance (Ex) of PBDE concentrations in soil and dusts across various e-waste sites compared to control sites in Lagos.

	Top Soil 0–10cm							Floor Dust (Control Soil)				Roadside Dust	
**PBDE**	**Control**	**Burning Sites**		**Dismantling Sites**		**Repair Sites**		**Dismantling Sites**		**Repair Sites**		**Dismantling Sites**	
**Title**	**Median**	**Median**	**Ex**	**Median**	**Ex**	**Median**	**Ex**	**Median**	**Ex**	**Median**	**Ex**	**Median**	**Ex**
BDE-28	0.01	40.54	4054	0.96	96.0	0.02	2.0	0.9	88.0	0.5	50.0	0.18	18.0
BDE-47	0.04	17.05	426	3.67	91.8	0.1	2.5	6.3	157.5	0.5	13.5	8.02	200.5
BDE-100	0.07	86.02	1229	1.4	20.0	0.31	4.4	4.1	58.6	0.4	5.7	4.44	63.4
BDE-99	0.22	158.11	719	10.83	49.2	0.53	2.4	15	68.6	1.8	8.2	19	86.4
BDE-154	0.06	80.25	1338	2.68	44.7	0.21	3.5	5.4	90.2	0.4	6.8	2.5	41.7
BDE-153	0.085	64.19	755	9.93	116.8	0.24	2.8	8.9	104.7	1.9	22.4	8.2	96.5
BDE-138	0.001	3.24	3240	0.14	140.0	0	0.0	0.82	820.0	0.1	120.0	0.29	290.0
BDE-183	0.08	153.73	1922	11.41	142.6	0.2	2.5	17.6	219.6	5.0	62.5	10.76	134.5
BDE-190	0.08	164.12	2052	4.31	53.9	0.19	2.4	3.3	41.3	0.4	5.5	1.19	14.9
BDE-208	0.14	497.38	3553	8.66	61.9	0.1	0.7	6.33	45.2	3.0	21.4	3.28	23.4
BDE-207	0.29	952.29	3284	21.03	72.5	0.17	0.6	18.8	64.7	5.7	19.7	9.37	32.3
BDE-206	0.15	626.79	4179	8.72	58.1	0.07	0.5	15	100.7	2.8	18.7	9.49	63.3
BDE-209	2.1	17587	8375	1491	710.0	0.85	0.4	3009	1432.9	432.7	206.1	961	457.6
$\sum_{13}$PBDE	**3.3**	20,431	6191	**1568**	475	**3**	0.9	3110	942.4	**451.4**	136.8	**1051**	318.5
pH	**8.04**	**7.7**		**7.89**		**7.87**		**8.4**		**8.31**		**8.29**	
TOC	**3.13**	**36.3**		**7.83**		**1.51**		**4.9**		**3.35**		**4.43**	

Table S3. PBDE median concentrations (ng/g dry weight) across various e-waste sites; and exceedance (Ex) of PBDE concentrations in soil and dusts across various e-waste sites compared to control sites in Ibadan.

	**Top Soil 0–10cm**					**Floor Dust (Control Soil)**		**Direct Dust**			
**PBDE**	**Control**	**Burning Sites**		**Dismantling Sites**		**Repair Sites**		**Dismantling Sites**		**Repair Sites**	
	**Median**	**Median**	**Ex**	**Median**	**Ex**	**Median**	**Ex**	**Median**	**Ex**	**Median**	**Ex**
BDE-28	0.03	18.5	617	0.04	1.33	1.03	34.3	1.24	41.3	3.8	127
BDE-47	0.12	83	693	0.2	1.7	4.12	34.3	64.6	538.33	14.62	122
BDE-100	0.1	20	200	0.17	1.7	1.51	15.1	24.2	242	4.8	48
BDE-99	0.27	107	396.3	0.42	1.6	10.64	39.41	151.2	560	26	95.2
BDE-154	0.09	22.5	250	0.1	1.1	1.61	18	12.4	138	4.3	47.8
BDE-153	0.17	60	363.6	0.5	3.03	7.5	45.5	33.1	201	12.4	75.15
BDE-138	0	3.5	0	0		0.43		1.73		1.05	
BDE-183	0.07	38.7	553	1.2	17.14	14.5	206.57	16.7	238.6	15.5	221
BDE-190	0.03	32.4	1080	0.1	3.33	1.5	50	2.6	87	4.9	163.3
BDE-208	0.15	174	1157	1.2	8	25	164.7	14.2	94.5	25.3	168.3
BDE-207	0.27	367	1360	1.9	7.04	78.4	290.37	31.7	117.4	42	155.6
BDE-206	0.19	120	632	0.4	2.11	65	342	31.7	167	33.44	176
BDE-209	9	5850	650	13	1.44	8043	893.7	3181	353.	1464	162.7
$\sum_{13}$PBDE	10.4	6895	660	18	1.7	8272	792	3566	342	1618	155
pH	**8.2**	**8.19**		**5**		**8.8**		**-**		**9**	
TOC	**3.6**	**16.78**		**5.8**		**5.29**		**-**		**18.56**	

Table S4. PBDE median concentrations (ng/g dry weight) across various e-waste sites; and exceedance (Ex) of PBDE concentrations in soil and dusts across various e-waste sites compared to control sites in Aba.

Top Soil 0–10cm (Control Roadside Dust)				Floor Dust (Control Roadside Dust)		Roadside Dust (Control Roadside Dust)		Title
**PBDE**	**Control**	**Burning sites**		**Repair Sites**		**Repair Sites**		**Control**
	**Median**	**Median**	**Ex**	**Median**	**Ex**	**Median**	**Ex**	
BDE-28	0.02	2.3	115	0.16	8	1.44	72.00	0.02
BDE-47	0.47	5.6	11.91	1.42	3.02	3.9	8.30	0.47
BDE-100	0.18	1.64	9.11	0.38	2.11	0.6	3.33	0.18
BDE-99	1.08	11.7	10.83	2.55	2.36	5.84	5.41	1.08
BDE-154	0.13	7.74	59.54	0.43	3.31	2.46	18.92	0.13
BDE-153	0.23	20.7	90.00	1.6	6.96	7.9	34.35	0.32
BDE-138	0.02	1.6	80.00	0.1	5.00	0.54	27.00	0.02
BDE-183	0.33	13.4	40.61	8.72	26.42	4.56	13.82	0.33
BDE-190	0.07	7.5	107.14	0.37	5.29	2.82	40.29	0.07
BDE-208	0.57	4.4	7.72	3.4	5.96	2.57	4.51	0.57
BDE-207	1.13	8.74	7.73	10	8.85	3.46	3.06	1.13
BDE-206	1.2	3.63	3.03	6	5.00	2.15	1.79	1.1
BDE-209	71.5	103.3	1.44	534	7.47	69	0.97	71.5
$\sum_{13}$PBDE	77.5	192	2.5	553	7.1	106	1.4	77.5
pH		**7.52**		**8.11**		**8.1**		**8.17**
TOC		**16.11**		**2.46**		**1.5**		**1.44**

Table S5. F-values of the two-Way ANOVA for PBDE concentration in all sample types across the Activity sites (burning. dismantling. repair sites).

PBDEs	Soil	Floor Dust	Roadside Dust	Direct Dust
	**F(df) = value Buring Dismantling and Control**	**T-value t(df) = value Dismantling and Repair Sites**	**T-value t(df) = value Dismantling and Control Sites**	**T-value t(df) = value Dismantling and Repair Sites**
**BDE-28**	(3.17) = 1.122	(27) = 1.366	(10) = 0.327 ***	(4) = −0.694
**BDE-47**	(3.17) = 3.467 *	(27) = 3.728	(10) = 4.267 ***	(4) = 0.862
**BDE-100**	(3.17) = 3.529 *	(27) = 5.401	(10) = 5.260 ***	(4) = 2.886*
**BDE-99**	(3.17) = 4.318 *	(27) = 4.314	(10) = 4.390 ***	(4) = −1.217
**BDE-154**	(3.17) = 5.348 **	(27) = 4.886	(10) = 6.171 ***	(4) = 2.214
**BDE-153**	(3.17) = 5.130 **	(27) = 0.988	(10) = 6.565 ***	(4) = 0.394
**BDE-138**	(3.17) = 5.708 **	(27) = 3.058	(10) = 3.154 **	(4) = 0.830
**BDE-183**	(3.17) = 6.336 **	(27) = 0.525	(10) = 5.841 ***	(4) = −0.079
**BDE-190**	(3.17) = 4.910 *	(27) = 3.716	(10) = 6.017 ***	(4) = −0.373
**BDE-208**	(3.17) = 6.438 **	(27) = 0.617	(10) = 3.308 **	(4) = −0.565
**BDE-207**	(3.17) = 6.038 **	(27) = 0.380	(10) = 3.555 **	(4) = −0.445
**BDE-206**	(3.17) = 3.502 *	(27) = 0.195	(10) = 2.387 *	(4) = −0.405
**BDE-209**	(3.17) = 4.796 *	(27) = 0.159	(10) = 2.543 *	(4) = −0.137
**$\sum_{13}$PBDE**				

*** = $p < 0.001$. ** = $p < 0.01$. * = $P < 0.05$.

Table S6. F-values of the two-Way ANOVA for PBDE concentration in top soils across the locations(Lagos. Ibadan. Aba) and Activity sites (burning. dismantling. repair. and control sites).

PBDEs	Activity Sites (ŋ)	Location (ŋ)	Activity site # location (ŋ)
BDE-28	21620.752 (1.000) ***	29885.907 (1.000) ***	29739.812 (1.000) ***
BDE-47	3.066 (0.414) *	0.361 (0.053)	0.871 (0.118)
BDE-100	5.026 (0.537) **	1.829 (0.220)	0.615 (0.086)
BDE-99	3.656 (0.458) *	0.197 (0.029)	0.625 (0.088)
BDE-154	6.676 (0.606) **	1.603 (0.198)	1.027 (0.136)
BDE-153	5.673 (0.567) **	0.827 (0.113)	1.230 (0.159)
BDE-138	4.764 (0.524) **	0.493 (0.070)	0.367 (0.053)
BDE-183	8.654 (0.666) ***	2.039 (0.239)	0.599 (0.084)
BDE-190	7.084 (0.620) **	2.520 (0.279)	0.989 (0.132)
BDE-208	11.822 (0.732) ***	4.062 (0.385) *	0.984 (0.131)
BDE-207	11.728 (0.730) ***	4.125 (0.388) *	1.180 (0.154)
BDE-206	5.706 (0.568) **	2.406 (0.270)	1.062 (0.140)
BDE-209	10.025 (0.698) ***	3.251 (0.333)	2.845 (0.304)
$\sum_{13}$PBDE	9.886 (0.695) ***	3.160 (0.327)	2.539 (0.281)

*** = $p < 0.001$. ** = $p < 0.01$. * =p <0.05. ŋ = partial Eta squared. # = interaction effect

Table S7. F-values of the two-Way ANOVA for PBDE concentration in floor dust across the locations (Lagos. Ibadan. Aba) and Activity sites (dismantling and repair sites).

PBDEs	Activity Sites (ŋ)	Location (ŋ)	Activity Site # Location (ŋ)
BDE-28	0.501 (0.020)	2.855 (0.192)	0.009 (0.000)
BDE-47	24.776 (0.508) ***	8.215 (0.406) **	0.759 (0.031)
BDE-100	30.994 (0.564) ***	3.961 (0.248) *	2.165 (0.083)
BDE-99	27.732 (0.536) ***	7.135 (0.373) **	1.832 (0.071)
BDE-154	22.493 (0.484) ***	3.262 (0.214)	5.311 (0.181) *
BDE-153	0.642 (0.026)	1.344 (0.101)	0.154 (0.006)
BDE-138	6.639 (0.217) **	2.189 (0.154)	0.940 (0.038)
BDE-183	0.013 (0.001)	0.832 (0.065)	2.074 (0.080)
BDE-190	8.460 (0.261) **	1.401 (0.105)	4.462 (0.157)
BDE-208	0.004 (0.000)	1.853 (0.134)	2.982 (0.111)
BDE-207	0.027 (0.001)	1.680 (0.123)	3.319 (0.122)
BDE-206	0.015 (0.001)	1.742 (0.127)	2.430 (0.092)
BDE-209	0.673 (0.008)	0.284 (0.023)	2.642 (0.99)
$\sum_{13}$PBDE	0.124 (0.005)	0.315 (0.026)	2.471 (0.093)

*** = $p < 0.001$. ** = $p < 0.01$. * = $p < 0.05$. ŋ= partial Eta squared. # = interaction effect

Table S8. Correlations between PBDEs congeners. ∑13PBDE. pH and TOC (Top soils).

PBDE congeners	BDE28	BDE47	BDE100	BDE99	BDE154	BDE153	BDE138	BDE183	BDE190	BDE208	BDE207	BDE206	BDE209	pH	TOC
BDE28	1													0.261	0.528
BDE47	0.702	1												0.296	0.569
BDE100	0.659	0.914	1											0.234	0.685
BDE99	0.706	0.972	0.973	1										0.295	0.530
BDE154	0.722	0.909	0.957	0.959	1									0.225	0.665
BDE153	0.691	0.816	0.811	0.845	0.879	1								0.126	0.291
BDE138	0.758	0.907	0.867	0.914	0.919	0.825	1							0.372	0.524
BDE183	0.713	0.803	0.791	0.828	0.848	0.893	0.871	1						0.198	0.510
BDE190	0.720	0.853	0.875	0.884	0.933	0.805	0.879	0.833	1					0.295	0.650
BDE208	0.775	0.819	0.796	0.843	0.828	0.824	0.824	0.880	0.817	1				0.192	0.597
BDE207	0.757	0.813	0.780	0.832	0.822	0.847	0.824	0.910	0.813	0.993	1			0.207	0.559
BDE206	0.726	0.825	0.726	0.803	0.758	0.785	0.805	0.864	0.760	0.951	0.962	1		0.261	0.482
BDE209	0.687	0.757	0.668	0.744	0.705	0.794	0.772	0.868	0.706	0.913	0.939	0.953	1	0.319	0.347

TOC = Total Organic matter content; ** correlation is significant at the 0.01 level(2-tailed); * correlation is significant at the 0.05 level(2-taile
